# Meta-analysis of the diagnostic value of ^18^F-FDG PET/CT in the recurrence of epithelial ovarian cancer

**DOI:** 10.3389/fonc.2022.1003465

**Published:** 2022-11-07

**Authors:** Xiaoyan Wang, Lifeng Yang, Yan Wang

**Affiliations:** ^1^ School of Nursing, Hexi University, Zhangye, China; ^2^ Peking University First Hospital Ningxia Women and Children's Hospital (Ningxia Hui Autonomous Region Maternal and Child Health Hospital), Yinchuan, China

**Keywords:** Epithelial ovarian cancer, recurrence, ^18^F-FDG PET/CT, diagnosis, meta-analysis

## Abstract

**Background:**

Ovarian cancer is the leading cause of cancer-related death among gynecologic malignancies. With much evidence suggesting that ^18^F-FDG PET/CT may be an excellent imaging test for the diagnosis of epithelial ovarian cancer recurrence, we conducted a systematic review and meta-analysis to summarize relevant studies and evaluate the accuracy and application value of ^18^F-FDG PET/CT in the diagnosis of recurrence of epithelial ovarian cancer.

**Materials and methods:**

Clinical trials of ^18^F-FDG PET/CT for the diagnosis of recurrence of epithelial ovarian cancer were systematically searched in PubMed, Embase, Cochrane Library, Web of Science and OVID database. The relevant literature was searched until May 22, 2022. Quality Assessment of Diagnostic Accuracy Studies (QUADAS-2) was used to evaluate the quality of the included original studies, and the meta-analysis was performed using a bivariate mixed-effects model and completed in Stata 15.0.

**Results:**

A total of 17 studies on ^18^F-FDG PET/CT for the diagnosis of epithelial ovarian cancer recurrence were included in this systematic review, involving 639 patients with epithelial ovarian cancer. Meta-analysis showed that the sensitivity, specificity and area under the curve of ^18^F-FDG PET/CT for the diagnosis of epithelial ovarian cancer recurrence were 0.88 (95% CI: 0.79 - 0.93), 0.89 (95% CI: 0.72 - 0.96) and 0.94 (95% CI: 0.91- 0.96), respectively. Subgroup analysis showed higher diagnostic efficacy in prospective studies than in retrospective studies, and no significant publication bias was observed in Deeks’ funnel plot, with sensitivity analysis revealing the stability of results. Meta regression shows that the heterogeneity of this study comes from study type.

**Conclusion:**

^18^F-FDG PET/CT has good diagnostic value in the recurrence of epithelial ovarian cancer.

## 1 Introduction

Ovarian cancer ranks as the eighth leading gynecologic malignancy in terms of both incidence and cause of death in women (1), with more than 220,000 cases of ovarian cancer and approximately 160,000 cancer-related deaths worldwide in 2010 (2, 3). Epithelial ovarian cancer is the most common pathological type of ovarian cancer with the highest mortality, accounting for about 85-90% of ovarian malignancies (4, 5), and 70% of patients with epithelial ovarian cancer have a 5-year survival rate of less than 30% and an extremely poor prognosis due to the diagnosis at an advanced stage (6). Although surgery and first-line chemotherapy improve the prognosis of epithelial ovarian cancer to some extent, 70-80% of patients still experience recurrence. The recurrence rate is 23% within 6 months after the end of the initial chemotherapy and up to 60% after 6 months (7). Disease recurrence is an important factor affecting clinical decision-making and survival prognosis of patients with epithelial ovarian cancer. Therefore, the search for imaging methods to accurately diagnose the recurrence of epithelial ovarian cancer has become an urgent clinical problem at present. Fluorine-18 fluorodeoxyglucose (FDG) positron emission tomography/computed tomography (^18^F-FDG PET/CT) is an imaging method with increasing relevance in this clinical context.

A previous meta-analysis revealed that FDG PET showed good sensitivity and moderate specificity, which may be a potentially effective method for identifying recurrent ovarian cancer (8). In order to update this meta-analysis and perform additional calculations, such as the area under the curve, a new meta-analysis on this topic was carried out. An international guideline on FDG PET/CT in ovarian cancer was published in 2021 by the European Association of Nuclear Medicine (EANM) and other international associations. It concluded that ^18^F-FDG PET/CT is effective in initial ovarian cancer detection, disease staging, and outcome determination, especially with class I evidence for recurrence detection (9).

In recent years, ^18^F-FDG PET/CT has gradually attracted attention for diagnosis and recurrence assessment in gynecologic tumors (10, 11). It also has significant advantages in the diagnosis of epithelial ovarian cancer recurrence due to its function of simultaneously providing information on glucose metabolism of tumor cells and anatomical structure of tumor lesions, as well as detection of systemic metastases in a single imaging session (12, 13).

Currently, there have been several international studies on the diagnosis of epithelial ovarian cancer recurrence by ^18^F-FDG PET/CT, whereas the conclusions are not consistent. Hence, we conducted this systematic review and meta-analysis to assess the overall diagnostic value of ^18^F-FDG PET/CT for the recurrence of epithelial ovarian cancer and to provide a reference for the clinical application of ^18^F-FDG PET/CT.

## 2 Materials and methods

### 2.1 Registration

We conducted this meta-analysis according to the guidelines of the Preferred Reporting Items for Systematic Reviews and Meta-Analyses (PRISMA) (14).

### 2.2 Search strategy

Studies on the diagnostic value of ^18^F-FDG PET/CT for epithelial ovarian cancer were searched in PubMed, Embase, Web of Science, OVID and Cochrane Library. Subject terms combined with free words were used as search criteria (MeSH in PubMed and Emtree in Embase), with the search period up to May 22, 2022.

We also reviewed references of relevant reviews and meta-analyses to identify eligible studies. The specific search strategy is described in Appendix 1. In addition, we supplemented the manual search to find relevant literature.

### 2.3 Literature selection criteria

Inclusion criteria: (a) the study included patients have diagnosed or suspected epithelial ovarian cancer. Additionally, they are suspected to suffer from recurrence and metastasis more than six months after the preoperative imaging examination, or six months after the first cytoreductive surgery and standard chemotherapy for ovarian cancer achieved clinical response; (b) the study used ^18^F-FDG PET/CT in the diagnosis of epithelial ovarian cancer recurrence; (c) the article was published in English.

Exclusion criteria: (a) letters, case reports, reviews, systematic review, meta-analysis and conference abstracts; (b) data that could not be extracted for true positives (TP), false positives (FP), false negatives (FN) and true negatives (TN); (c) duplicate studies.

### 2.4 Data extraction

We performed an initial search to remove duplicate records, filter titles and abstracts for relevance, and identify records for inclusion, exclusion, or uncertainty. For uncertain studies, the full text was obtained to determine whether the requirements were met. Data were extracted as follows: (1) first author, year of publication, country, number of recurrent patients, the total number of patients, age, type of study design, the dose of imaging agent used, and threshold; (2) outcome indicators of TP, FP, FN, and TN. Two investigators (WXY and YLF) performed data extraction independently, and the third investigator (WY) was consulted to resolve discrepancies.

### 2.5 Data abstraction

As in a previous meta-analysis by Delgado-Bolton et al. (15), on the basis of their design, 2 types of studies were differentiated; in some, ^18^F-FDG PET CT was performed when epithelial ovarian cancer was diagnosed recurrence(type I); whereas, in others (type II), ^18^F-FDG PET CT was compared with CT or MRI or tumor markers or ultrasound in related study that included epithelial ovarian cancer patients who had presented results for recurrence detection in all of the following diagnostic procedures (if performed in each particular patient): (a) careful clinical history and complete physical examination; (b) laboratory analysis(tumor markers); (c) radiologic or isotopic procedures except ^18^F-FDG PET CT, CT, MRI, and ultrasound; or even (d) surgical exploration, biopsy, or fine-needle aspiration cytology of suspicious lesions.

### 2.6 Quality assessment

The methodological quality of the selected eligible articles was assessed using the Quality Assessment of Diagnostic Accuracy Studies 2 (QUADAS-2) scoring system. Two authors (WXY and YLF) independently assessed the methodological quality of the included studies and, in case of disagreement, the third investigator (WY) was consulted to resolve the dispute.

### 2.7 Data analysis

Risk of bias assessment plots for the included studies were completed using RevMan 5.4 software. Stata 15 software (Stata Corp LP, College Station, TX, USA) was adopted to perform additional statistical analyses. Diagnostic threshold effects were evaluated using the typical “shoulder-arm shape” of the summary receiver operating characteristic (SROC) curve, and a bivariate mixed-effects model was used to analyze the data. Sensitivity (Sen), specificity (Spe), positive likelihood ratio (PLR), negative likelihood ratio (NLR), and diagnostic odds ratio (DOR) were combined, and the corresponding forest plots were drawn to calculate the area under the curve (AUC) values. Moreover, the inconsistency index (I^2^) and P-value were used to evaluate the heterogeneity among studies. Heterogeneity was considered significant when I^2^ > 50% or P > 0.05.

The relationship between the prior probability, likelihood ratio and posterior probability was evaluated using Fagan’s nomogram (16). Meta-regression and subgroup analysis were employed to analyze the sources of heterogeneity of the included studies, which were performed according to the design type (prospective or retrospective study), area (Asia or Europe or North America), study type (type I or type II). Sensitivity analysis was adopted to verify the robustness of the study results, and Deeks’ funnel plot was used to assess publication bias.

## 3 Results

### 3.1 Study selection

**Figure 1:**
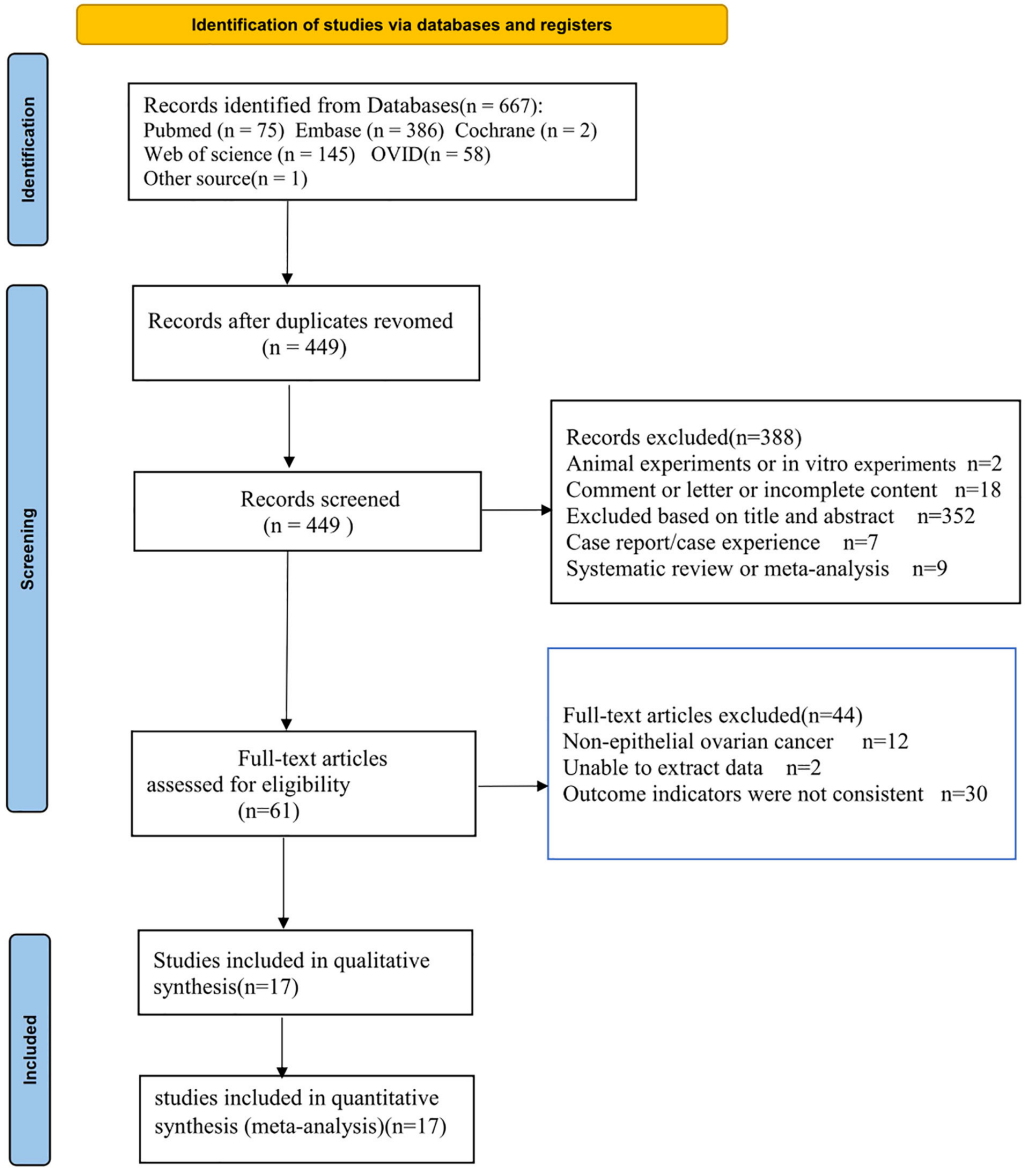
Flow diagram of studies selection process.

The flow chart of the selected studies is shown in **Figure 1**. A total of 667 articles were retrieved from the initial literature search, and 218 duplicate articles were removed. Of the remaining 449 articles, 388 records were excluded by title and abstract screening, and then the full text of 61 articles was screened, 44 of which were excluded. A total of 17 articles were eventually included in the meta-analysis (17–33).

### 3.2 Characteristics of the included studies

A total of 920 cases were included in the present study, and recurrence was confirmed in 639 patients by pathology and clinical follow-up. The basic characteristics and more details of the included literature are shown in **Table 1**. The literature was published from 2002 to 2021; four were prospective studies (17, 18, 22, 32) and thirteen were retrospective studies (19–21, 23–31, 33); eleven of the sample sources were in Asian countries (17, 20–22, 24–26, 28, 29, 32, 33), four in Europe (18, 19, 23, 27), and two in North America (30, 31); nine articles mentioned image use doses (17, 19–24, 27, 29–33) and only three addressed diagnostic thresholds (19, 28, 32). With regard to the types of studies described in the methods, 7 studies were type I (18, 22–24, 26, 30, 33), 10 studies were type II (17, 19–21, 25, 27–29, 31, 32).

**Table 1:** Basic information of the literature on ^18^F-FDG PET/CT for the diagnosis of epithelial ovarian cancer recurrence.

Author	Age	Study design	Period	Recurrence	Study type	Study area	TP	FP	FN	TN	Dose	Thresholdvalue
Nawi N M, 2021 (17)	16-73	PS	2011-2013	17	type II	Malaysia	16	2	1	24	370–740 mbq	
Vallius T, 2018 (18)	50-80	PS	2009-2014	10	type I	Finland	7	4	3	15	/	
Palomar Muñoz A, 2018 (19)	60.0 ± 12.6	RS	2007-2015	45	type II	Spain	45	0	0	13	370mbq	
Lee YJ, 2018 (20)	17-76	RS	2000-2013	65	type II	Korea	64	8	1	0	370–555 mbq	SUV max>3.0
Li M, 2017 (21)	33-72	RS	2005-2016	51	type II	China	49	4	2	6	5.5 mBq/kg	
Kim TH, 2017 (22)	42-75	PS	2020-2013	5	type I	Korea	4	0	1	8	0.15mCi/kg	
Gonzalez Garcia B, 2017 (23)	33-85	RS	2010-2015	30	type I	Spain	23	0	7	1	370mbq	
Chung HH, 2017 (24)	33-79	RS	2010-2014	57	type I	Korea	32	11	25	26	0.14 mCi/kg	
Ghosh J, 2013 (25)	40-75	RS	2006-2008	15	type II	India	15	0	0	1	/	
Chung HH, 2012 (26)	35-80	RS	2004-2009	20	type I	Korea	22	10	6	17	/	
Antunovic L, 2012 (27)	57. 0 ± 11.8	RS	/	97	type II	Italy	80	3	21	17	0.15-0.16Ci/kg	
Nasu K, 2011 (28)	32-78	RS	2006-2009	15	type II	Japan	18	0	4	8	/	
Jiajing XI, 2011 (29)	29-73	RS	2004-2010	34	type II	China	31	4	3	14	0.1mCi/kg	SUV max>2.5
Iagaru AH, 2008 (30)	27-80	RS	2003-2006	36	type I	America	31	1	5	6	381.1-769.6mbq	
García-Velloso MJ, 2007 (31)	49-63	RS	/	80	type II	Canada	80	7	12	26	370-400mbq	
Murakami M, 2006 (32)	35-76	PS	1997-2002	46	type II	Japan	42	0	4	44	370mbq	
Cho SM, 2002 (33)	17-70	RS	1996-2000	16	type I	Korea	7	0	9	15	370mbq	SUV max>3.0

PS, prospective study; RS, retrospective study;/, not described.

### 3.3 Quality assessment of included studies according to the QUADAS-2 guidelines

**Figure 2:**
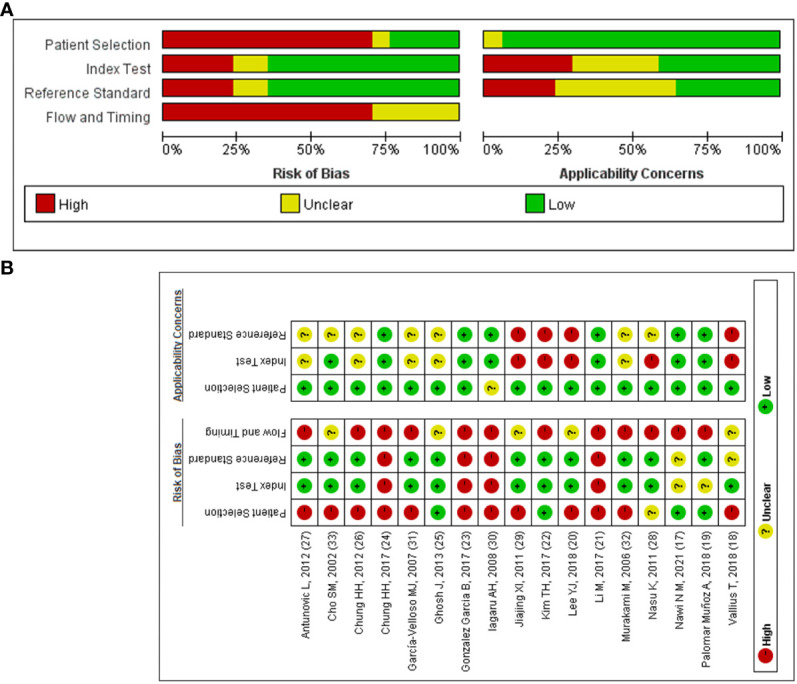
The quality of included articles according to the QUADAS-2 guidelines. **(A)** Risk of bias summary; **(B)** Risk of bias graph.

QUADAS-2 was used to assess the quality of the included literature (**Figures 2A, B**). As shown in **Figure 2B**, the quality of reporting on the selection of included cases, the gold standard, and the case flow and progression of cases is good, while the quality of reporting on the implementation of diagnostic tests is poor and uneven. This is mainly related to inappropriate inclusion of study subjects (18, 20, 21, 23, 24, 26, 27, 29–33), unclear implementation of literature blinding (17, 18, 20–33) and reporting of thresholds (17–19, 21–28, 30–32), and unclear risk of bias or high risk of bias in the time interval between the trial to be evaluated and the gold standard (18–33).

#### 3.3.1 Meta-analysis results of diagnostic accuracy and heterogeneity analysis

**Figure 3:**
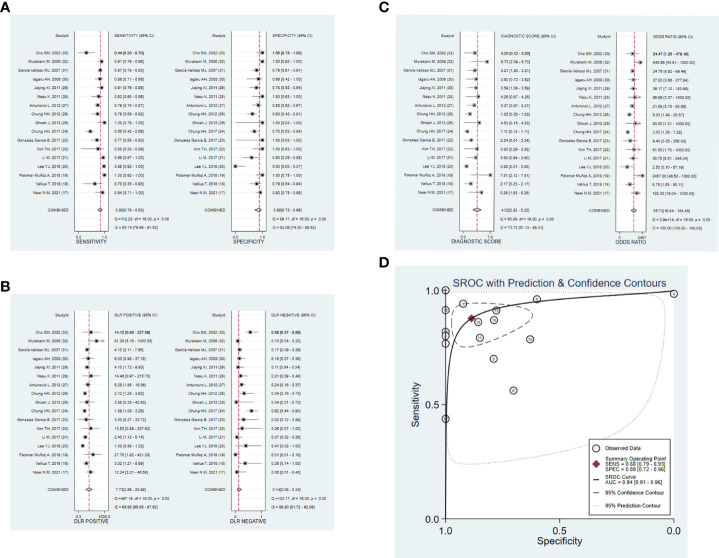
Forest plots of ^18^F-FDG PET/CT for the diagnostic value of epithelial ovarian cancer. **(A)** sensitivity and specificity. **(B)** PLR and NLR. **(C)** DOR. **(D)** SROC. DOR, diagnostic odds ratio; PLR, positive likelihood ratio; NLR, negative likelihood ratio; SROC, summary receiver operating characteristic.

**Figure 4:**
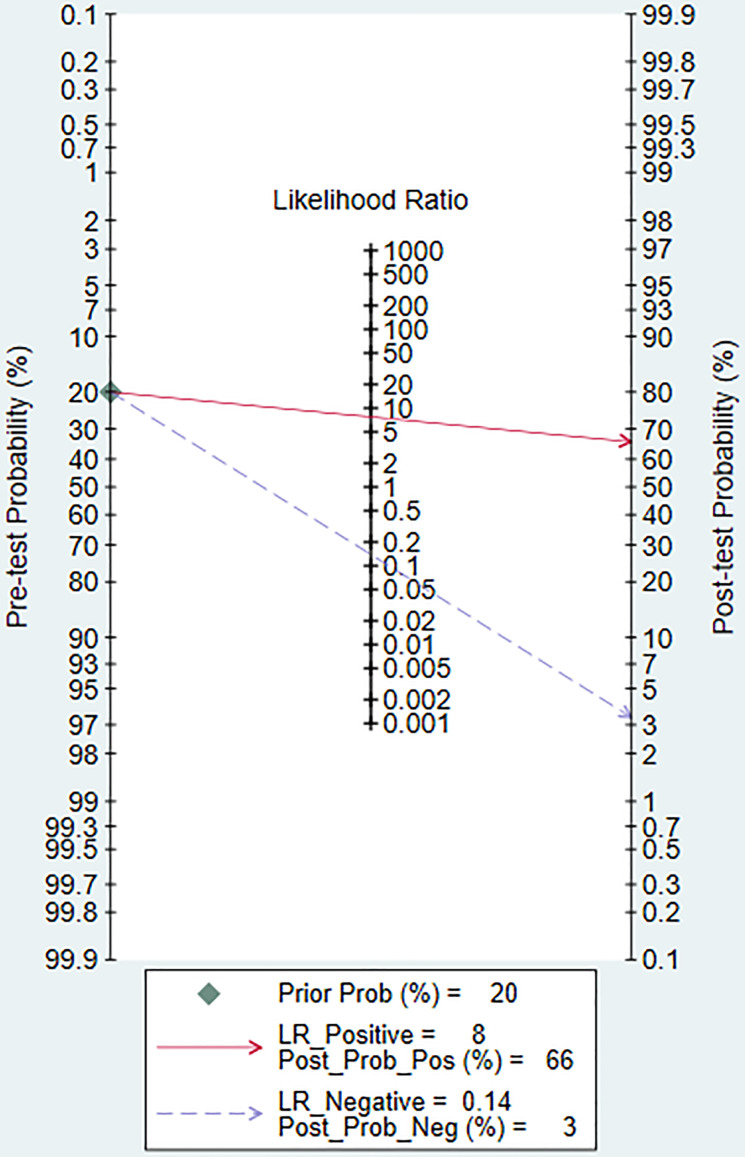
Fagan’s plot of PLR and NLR to evaluate the clinical utility of ^18^F-FDG PET/CT in the diagnosis of epithelial ovarian cancer. PLR, positive likelihood ratio; NLR, negative likelihood ratio.

**Figures 3 A–D** shows the forest plots and SROC curve of the diagnostic accuracy of ^18^F-FDG PET/CT. The SROC curve (**Figure 3D**) does not show the typical “shoulder-arm shape”, indicating that there is no threshold effect in the diagnostic test. The results of the bivariate mixed-effects model showed that the combined sensitivity (**Figure 3A**), specificity (**Figure 3A**), positive likelihood ratio (**Figure 3B**), negative likelihood ratio (**Figure 3B**), and diagnostic odds ratio (**Figure 3C**) were 0.88 (95% CI: 0.79-0.93), 0.89 (95% CI: 0.72-0.96), 7.73 (95% CI: 2.86-20.89), 0.14 (95% CI: 0.08-0.24), and 4.02 (95% CI: 2.82-5.22), respectively. In addition, the AUC value was 0.94 (95% CI: 0.91-0.96). The predictive probability plot of Fagan’s nomogram shows that if the pre-test probability ratio is 20%, the post-test probability is 66% for PLR (**Figure 4**)and 3% for NLR (**Figure 4**), suggesting the good value of ^18^F-FDG PET/CT for the diagnosis of epithelial ovarian cancer.

### 3.4 Exploration of sources of heterogeneity in included studies by meta-regression and subgroup analysis

**Figure 5:**
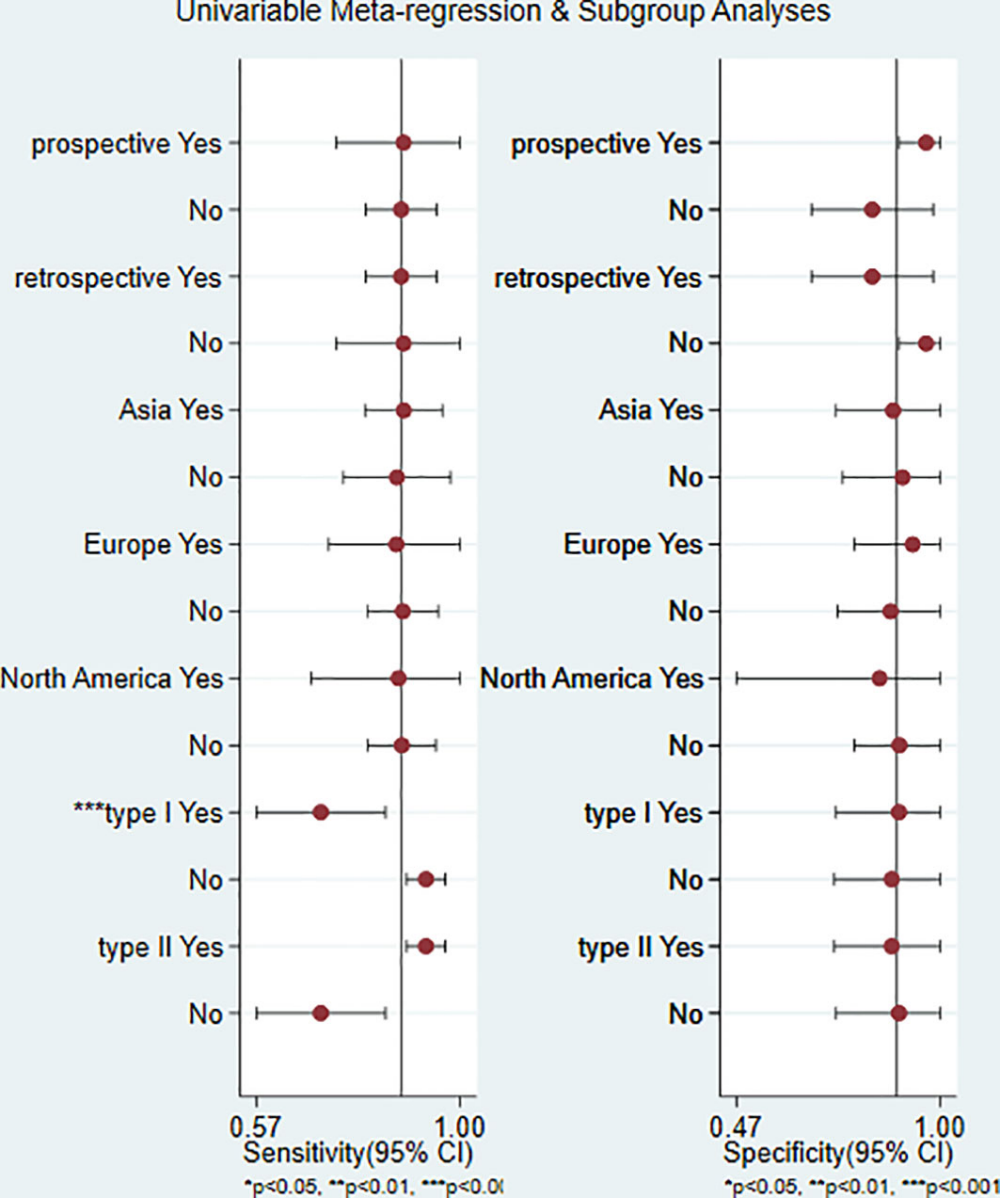
Meta-regression and subgroup analyses.

Design type, study area and study type were included in the regression model. There was no significant difference in the combined sensitivity and specificity of different design types and areas (*P* > 0.05). In the study type, the combined sensitivity of type I was statistically significant (*P* < 0.05). It is speculated that the heterogeneity of this study is from study type. Details of the meta-regression are shown in **Table 2** and **Figure 5**, and details of the subgroup analysis are shown in **Table 3**.

**Table 2:** Summary estimated of diagnostic performance of ^18^F-FDG PET/CT for epithelial ovarian cancer based on design type.

Subgroup	Study	Sen (95%CI)	Spe (95%CI)	PLR (95%CI)	NLR (95%CI)	DOR (95%CI)	AUC (95%CI)
Prospective study	4	0.87 (0.74, 0.94)	0.96 (0.78, 0.99)	19.87 (3.34, 118.13)	0.13 (0.06, 0.29)	5.00 (2.72, 7.28)	0.94 (0.92, 0.96)
Retrospective study	13	0.88 (0.77, 0.94)	0.82 (0.60, 0.93)	4.9 (2.0, 12.0)	0.15 (0.08, 0.28)	33.0 (11, 105)	0.92 (0.89, 0.94)

**Table 3:** Results of meta-regression analysis.

Parameter	Category	Number of study	Sensitivity (95%CI)	*P*	Specificity (95%CI)	*P*
Design type	Prospective	4	0.88 (0.74, 1.00)	0.60	0.96 (0.89, 1.00)	0.05
	Retrospective	13	0.88 (0.80, 0.95)	0.53	0.82 (0.67, 0.98)	0.20
Study area	Asia	11	0.88 (0.80, 0.96)	0.35	0.88 (0.73, 1.00)	0.98
	Europe	4	0.87 (0.73, 1.00)	0.37	0.93 (0.78, 1.00)	0.26
	North America	2	0.87 (0.69, 1.00)	0.77	0.84 (0.47, 1.00)	0.75
Study type	Type I	7	0.71 (0.57, 0.84)	0.00	0.89 (0.73, 1.00)	0.70
	Type II	10	0.93 (0.89, 0.97)	0.52	0.87 (0.73, 1.00)	0.97

### 3.5 Sensitivity analysis to verify the robustness of the study results

**Figure 6:**
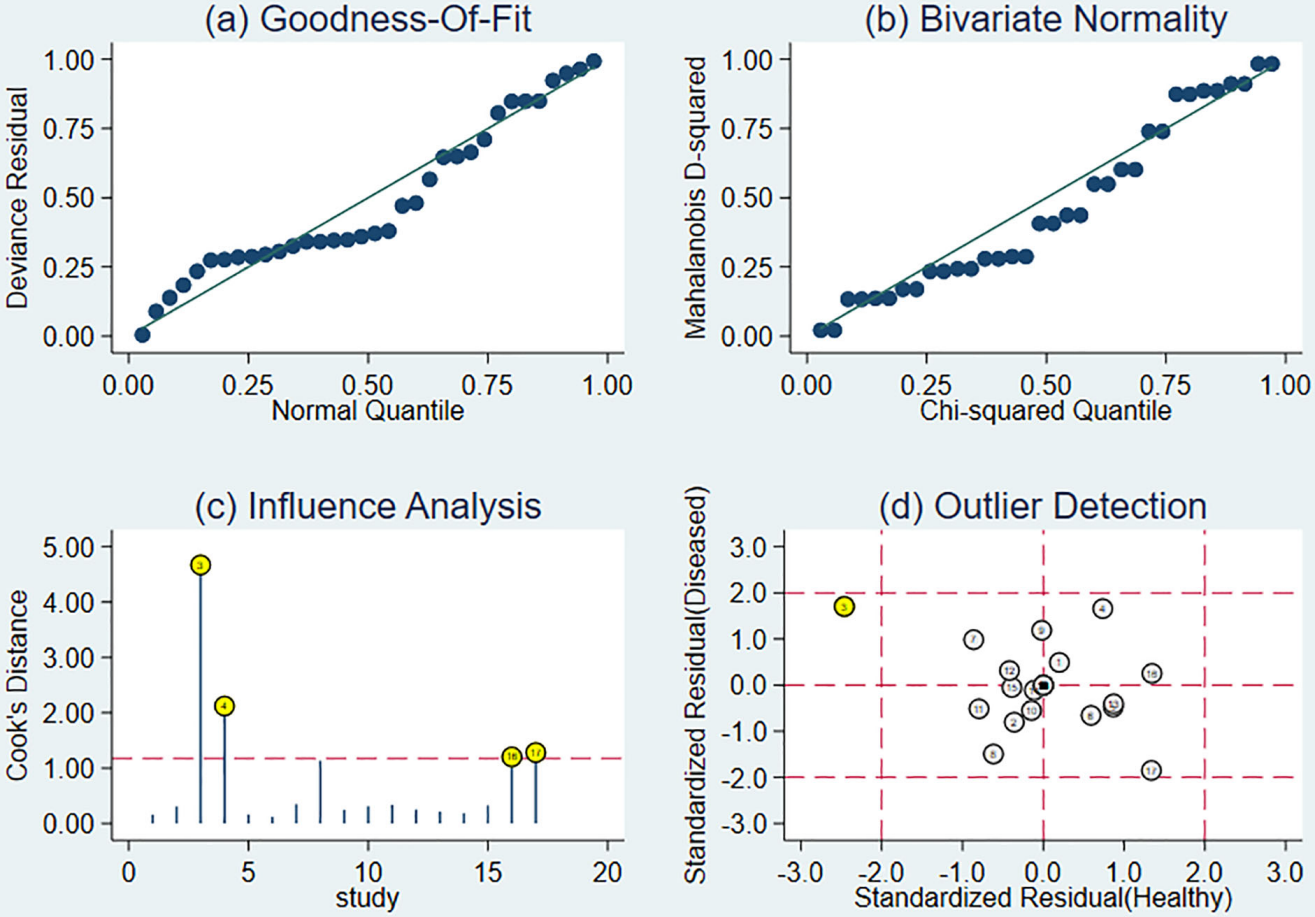
The results of sensitivity analysis. **(A)** Goodness-of-ft. **(B)** Bivariate normality. **(C)** Influence analysis. **(D)** Outlier detection.

Sensitivity analysis showed a good fit for the goodness of fit and binary normality (**Figure 6A, B**). There were four articles weighted (**Figure 6C**) (34), which may be a source of heterogeneity shown by outlier detection (**Figure 6D**). After the exclusion of abnormal studies, sensitivity decreased slightly from 0.88 to 0.85, specificity decreased from 0.89 to 0.80, AUC value decreased from 0.94 to 0.87, and DOR decreased from 4.02 to 3.12.

### 3.6 Detection of publication bias

**Figure 7:**
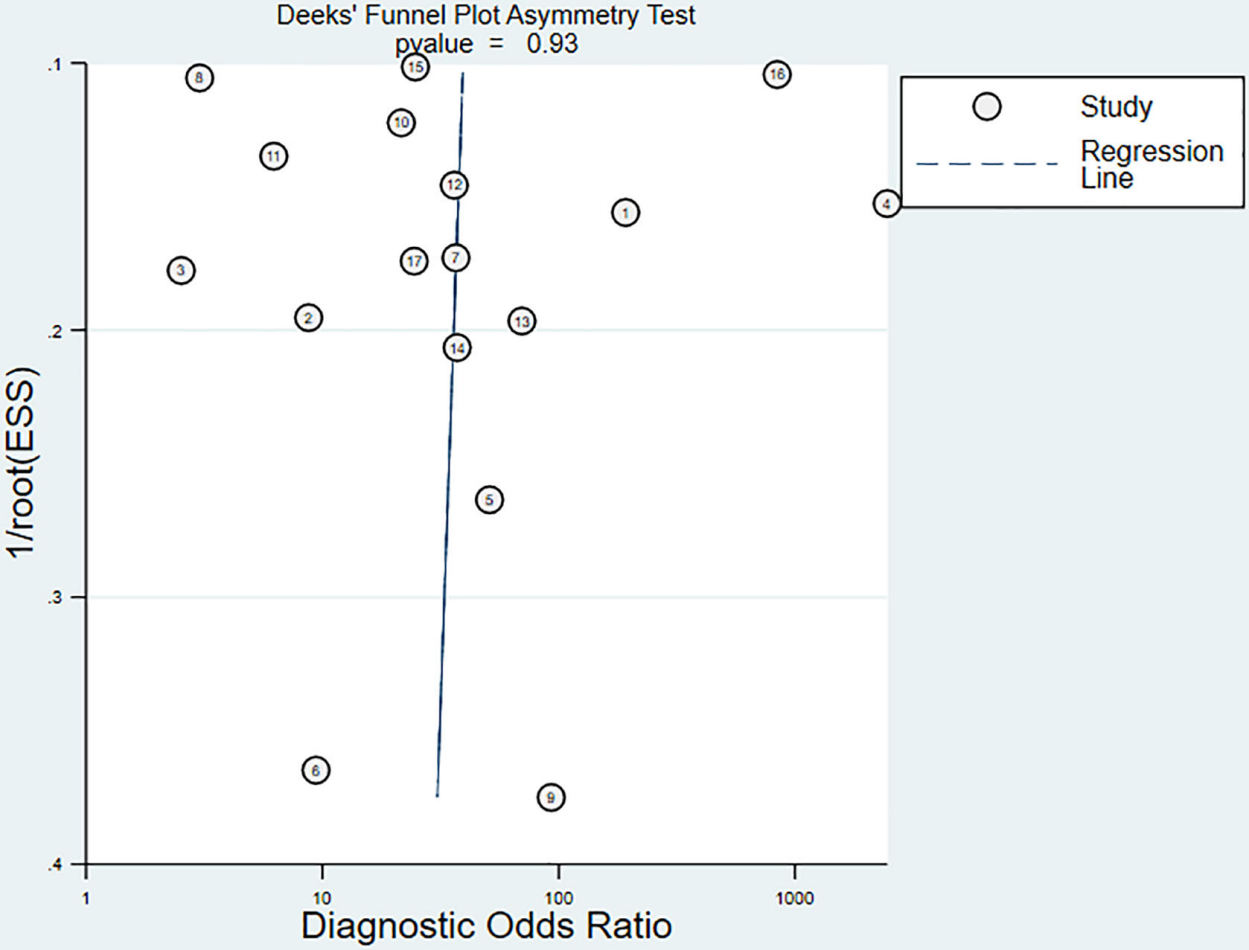
The Deeks’ funnel plot for assessing the publication bias.

The Deeks’ funnel plot showed a *P*-value of 0.93, indicating that there was no publication bias in this meta-analysis (**Figure 7**).

## 4 Discussion

With a total of 17 articles included in this study, the results showed that the sensitivity and specificity of ^18^F-FDG PET/CT in diagnosing recurrence of epithelial ovarian cancer were 88% and 89%, and the AUC value of ^18^F-FDG PET/CT in assessing recurrence of epithelial ovarian cancer was 0.94, suggesting its high diagnostic efficacy. Through reviewing previous meta-analyses, the AUC values for single ctDNA and microRNA to detect recurrence of epithelial ovarian cancer were 0.884 and 0.894 (35), and the AUC values for ROMA values and HE 4 to detect recurrence of epithelial ovarian cancer were 0.93 and 0.91, respectively (36, 37). In the combined serum tumor marker test, the diagnostic AUC value of the combined serum CA125, CA19.9 and CEA test was 0.92 (38), all of which were lower than the diagnostic ability of ^18^F-FDG PET/CT.

However, the false-negative rate (1-Sen) and misdiagnosis rate (1-Spe) of ^18^F-FDG PET/CT were 12% and 11%, respectively, suggesting that there were certain missed diagnoses and misdiagnoses in ^18^F-FDG PET/CT diagnosis. According to relevant literature and clinical experience, infection or inflammation is one of the common causes of misdiagnosis (17, 20). Strong uptake of the tracer ^18^F-FDG by anti-inflammatory cells such as macrophages or granulation tissue activated in areas of inflammation leads to active inflammatory lesions or abscesses that may be misinterpreted as malignant tumors. Other causes include ureteral stasis, atherosclerotic plaque, dislocations due to peristalsis, bladder filling or diverticulitis. Moreover, some benign gynecologic diseases and peritoneal disease may also lead to false-positive results of ^18^F-FDG PET/CT (39), including endometritis, follicular cyst, functional corpus luteum cyst, tubal oophoritis, fibroids, cystadenofibroma, teratoma, endometriosis, oviduct ovarian abscess, benign meningioma, schwannoma, and pelvic tuberculosis. Meanwhile, given the usually low FDG uptake of malignant peritoneal disease, it is recommended to study in detail both the CT component of the FDG PET/CT and previous contrast enhanced CTs to improve the accuracy (9, 40). Common reasons for missed findings are first, the location of the lesion is too close to the bladder, which contains high concentrations of ^18^F-FDG and may be confused with urinary secretions (41); second, the resolution of the lesion is low when it is in a hypometabolic state (42), and third, the size of the lesion can also affect the accuracy of diagnosis of ovarian cancer. Pannu et al. (43) reported a sensitivity of 50-90% for ^18^F-FDG PET/CT for the diagnosis of recurrent ovarian cancer lesions >1 cm (44) and 13% for lesions <1 cm (45). Therefore, in clinical practice, in addition to the causes of missed diagnosis and misdiagnosis, the possibility of reducing the rate of missed diagnosis and misdiagnosis by combining serum tumor marker detection should also be considered. The use of serum tumor marker testing alone is prone to false positives, while in the diagnosis of recurrence of ovarian cancer, the sensitivity of CA 125 and HE 4 combined with ^18^F-FDG PET/CT was increased to 98% - 100% (46), and the specificity of CA 125 combined with ^18^F-FDG PET/CT was increased to 91.8% (21). We recommend the use of ^18^F-FDG PET/C in combination with serum tumor markers for monitoring the recurrence of epithelial ovarian cancer.

Early diagnosis and treatment can improve the survival rate and prolong the survival time of ovarian cancer patients. At present, clinical diagnosis of epithelial ovarian cancer recurrence mainly relies on imaging examination and histopathological examination. Histopathological examination is the gold standard for the diagnosis of malignant tumors, but it has not been widely used in cancer diagnosis due to its invasiveness and high cost (47). The commonly used imaging examination methods are ultrasound, CT and MRI. Ultrasonography is simple and safe, but it is easily affected by the patient’s body shape, abdominal fluid, and abdominal wall thickness, leading to missed diagnoses. CT and MRI examinations provide an effective reference for accurate localization, measurement and efficacy evaluation of lesions, but the degree of detection of lesions by CT and MRI is proportional to the size of lesions. The imaging visibility is better when the lesions are more than 5-10mm, while the false negative rate increases when the diameter of the recurrence lesion in the pelvic cavity is less than 1 cm. Due to the susceptibility to physiological imaging of the intestinal and urinary systems, CT and MRI may also have some difficulties in differentiating abdominal and pelvic lesions from tumor recurrence (48, 49), and thus ordinary imaging examination should not be used as the primary examination to determine the recurrence of epithelial ovarian cancer.

PET is a clinical molecular imaging test that can detect positron-emitting radionuclides. It uses positron nuclides or positron nuclide-labeled compounds (such as ligands, antibodies, and enzymes) as tracers to detect the equal and opposite γ-ray produced by the combination and annihilation of positron and negative electrons in the living body (50). PET-CT is a PET-based integrated device that utilizes the X-ray CT in the same device to perform attenuation correction on the γ-ray detected by PET. In this way, it can provide high-resolution anatomical structure information of molecular-level tissue cells obtained by PET. Meanwhile, it can not only fuse PET and CT images but also offer independent CT diagnosis function (51). As PET-CT equipment keeps updating and improving, now it can detect specific physiological components inside the human body. Therefore, PET-CT has been widely recognized as one of the most advanced clinical molecular imaging equipment.

Ovarian tumor imaging usually uses 18 FDG as a tracer, whose molecular structure is similar to glucose. After entering the body, it is taken up by cells through the glucose transport mechanism and retained in cells but will not be further metabolized, and the glucose metabolism rate of ovarian malignant tumors is significantly higher than that of normal tissues. Compared with other imaging examinations, ^18^F-FDG PET/CT can more accurately display the concentration of imaging agent uptake and the metabolism and recurrence of the imaging agent in the lesion. Meanwhile, ^18^F-FDG PET/CT imaging is superior to other imaging examinations in diagnosing ovarian cancer metastases because of the lower location of the ovary in the pelvis, less fusion error between PET and CT images, and less interference by respiratory motion during imaging (52–54). Relevant studies have also demonstrated (27, 55) that the diagnostic value of ^18^F-FDG PET/CT for suspected epithelial ovarian cancer recurrence is higher than that of conventional imaging examination.

Furthermore, the results of subgroup analysis and meta-regression analysis showed that the diagnostic efficacy of ^18^F-FDG PET/CT for the assessment of epithelial ovarian cancer recurrence was less influenced by study design, study area and study type. Meta regression shows that the heterogeneity of this study comes from study type, which is caused by differences between studies. Subgroup analysis of our results based on different study designs found that ^18^F-FDG PET/CT was more effective in the prospective study than the retrospective study in the diagnosis of epithelial ovarian cancer recurrence, with an AUC value of 0.94 (95%CI: 0.92-0.96), the sensitivity of 0.87 and specificity of 0.96, indicating that the diagnostic ability of this interval was higher than that of other intervals.

This study also has the following limitations which need to be improved: first, most of the studies included in this study were retrospective, thus preventing an accurate evaluation of the blinding method; second, the 17 included studies had different follow-up times and doses of imaging agents used, and only three articles addressed the threshold for ^18^F-FDG PET/CT use, leading to possible heterogeneity.

## 5 Conclusion

In conclusion, our analysis suggests that the overall diagnostic value and accuracy of ^18^F-FDG PET/CT for recurrence in patients with epithelial ovarian cancer may be quite high. It can be used as an effective imaging method to diagnose the recurrence of epithelial ovarian cancer. We look forward to further studies to confirm our analysis.

## Data availability statement

The original contributions presented in the study are included in the article/**Supplementary Material**. Further inquiries can be directed to the corresponding author.

## Author contributions

XW and LY conceived and designed the study. XW and LY selected the studies and collected the data. XW analyzed data. All authors interpreted the results. YW drafted the paper. All authors revised the draft paper. All authors contributed to the article and approved the submitted version.

## Funding

This study was supported by the Fifth Ningxia Youth Science and Technology Talents Project Acknowledgments.

## Conflict of interest

The authors declare that the research was conducted in the absence of any commercial or financial relationships that could be construed as a potential conflict of interest.

## Publisher’s note

All claims expressed in this article are solely those of the authors and do not necessarily represent those of their affiliated organizations, or those of the publisher, the editors and the reviewers. Any product that may be evaluated in this article, or claim that may be made by its manufacturer, is not guaranteed or endorsed by the publisher.
