# Supplementary material for: Meta-analysis of the diagnostic value of 18F-FDG PET/CT in the recurrence of epithelial ovarian cancer
Source: Front Oncol. 2022 Nov 7;12:1003465. doi: 10.3389/fonc.2022.1003465 (PMC9676502; doi:10.3389/fonc.2022.1003465)
Supplement: Supplementary file 1 [file Table_1.docx]

Embase

| Search number | Query | Results |
| --- | --- | --- |
| #1 | 'ovary carcinoma'/exp | 38884 |
| #2 | 'carcinomas, ovarian epithelial':ti,ab,kw OR 'epithelial carcinoma, ovarian':ti,ab,kw OR 'epithelial carcinomas, ovarian':ti,ab,kw OR 'ovarian epithelial carcinomas':ti,ab,kw OR 'epithelial ovarian cancer':ti,ab,kw OR 'ovarian epithelial cancer':ti,ab,kw OR 'cancer, ovarian epithelial':ti,ab,kw OR 'cancers, ovarian epithelial':ti,ab,kw OR 'epithelial cancer, ovarian':ti,ab,kw OR 'epithelial cancers, ovarian':ti,ab,kw OR 'ovarian epithelial cancers':ti,ab,kw OR 'ovarian cancer, epithelial':ti,ab,kw OR 'cancer, epithelial ovarian':ti,ab,kw OR 'cancers, epithelial ovarian':ti,ab,kw OR 'epithelial ovarian cancers':ti,ab,kw OR 'ovarian cancers, epithelial':ti,ab,kw OR 'ovarian epithelial carcinoma':ti,ab,kw OR 'epithelial ovarian carcinoma':ti,ab,kw OR 'carcinoma, epithelial ovarian':ti,ab,kw OR 'carcinomas, epithelial ovarian':ti,ab,kw OR 'epithelial ovarian carcinomas':ti,ab,kw OR 'ovarian carcinoma, epithelial':ti,ab,kw | 18834 |
| #3 | #1 OR #2 | 46273 |
| #4 | 'fluorodeoxyglucose f 18'/exp | 69256 |
| #5 | 'f18, fluorodeoxyglucose':ti,ab,kw OR '18f fdg':ti,ab,kw OR 'fluorodeoxyglucose f 18':ti,ab,kw OR 'f 18, fluorodeoxyglucose':ti,ab,kw OR 'fludeoxyglucose f 18':ti,ab,kw OR 'f 18, fludeoxyglucose':ti,ab,kw OR 'fluorine 18 fluorodeoxyglucose':ti,ab,kw OR '18f fluorodeoxyglucose':ti,ab,kw OR 'fluorodeoxyglucose, 18f':ti,ab,kw OR 18fdg:ti,ab,kw OR '2 fluoro 2 deoxy d glucose':ti,ab,kw OR '2 fluoro 2 deoxyglucose':ti,ab,kw | 23631 |
| #6 | #4 OR #5 | 46273 |
| #7 | #3 AND #6 | 386 |

Cochrane library

| Search number | Query | Results |
| --- | --- | --- |
| #1 | " Carcinoma, Ovarian Epithelial"[Mesh] | 298 |
| #2 | (Carcinomas, Ovarian Epithelial):ti,ab,kw OR (Epithelial Carcinoma, Ovarian):ti,ab,kw OR (Epithelial Carcinomas, Ovarian):ti,ab,kw OR (Ovarian Epithelial Carcinomas):ti,ab,kw OR (Epithelial Ovarian Cancer):ti,ab,kw | 2115 |
| #3 | (Ovarian Epithelial Cancer):ti,ab,kw OR (Cancer, Ovarian Epithelial):ti,ab,kw OR (Cancers, Ovarian Epithelial):ti,ab,kw OR (Epithelial Cancer, Ovarian):ti,ab,kw OR (Epithelial Cancers, Ovarian):ti,ab,kw | 2006 |
| #4 | (Ovarian Epithelial Cancers):ti,ab,kw OR (Ovarian Cancer, Epithelial):ti,ab,kw OR (Cancer, Epithelial Ovarian):ti,ab,kw OR (Cancers, Epithelial Ovarian):ti,ab,kw OR (Epithelial Ovarian Cancers):ti,ab,kw | 2006 |
| #5 | (Ovarian Cancers, Epithelial):ti,ab,kw OR (Ovarian Epithelial Carcinoma):ti,ab,kw OR (Epithelial Ovarian Carcinoma):ti,ab,kw OR (Carcinoma, Epithelial Ovarian):ti,ab,kw OR (Carcinomas, Epithelial Ovarian):ti,ab,kw | 2115 |
| #6 | (Epithelial Ovarian Carcinomas):ti,ab,kw OR (Ovarian Carcinoma, Epithelial):ti,ab,kw | 1111 |
| #7 | #1 OR #2 OR #3 OR #4 OR #5 OR #6 | 2115 |
| #8 | " Fluorodeoxyglucose F18"[Mesh] | 643 |
| #9 | (F18, Fluorodeoxyglucose):ti,ab,kw OR (18F-FDG):ti,ab,kw OR (Fluorodeoxyglucose F 18):ti,ab,kw OR (F 18, Fluorodeoxyglucose): ti,ab,kw OR (Fludeoxyglucose F 18) | 1333 |
| #10 | (18FDG):ti,ab,kw OR ("2-Fluoro-2-deoxy-D-glucose"):ti,ab,kw OR (2 Fluoro 2 deoxy D glucose):ti,ab,kw OR ("2-Fluoro-2-deoxyglucose"):ti,ab,kw OR (2 Fluoro 2 deoxyglucose):ti,ab,kw | 328 |
| #11 | (F 18, Fludeoxyglucose):ti,ab,kw OR ("Fluorine-18-fluorodeoxyglucose"):ti,ab,kw OR (Fluorine 18 fluorodeoxyglucose):ti,ab,kw OR (18F Fluorodeoxyglucose):ti,ab,kw OR (Fluorodeoxyglucose, 18F):ti,ab,kw | 732 |
| #12 | #8 OR #9 OR #10 OR #11 | 1622 |
| #13 | #7 AND #12 | 2 |

Web of science

| Search number | Query | Results |
| --- | --- | --- |
| #1 | TS=(Carcinoma, Ovarian Epithelial OR Carcinomas, Ovarian Epithelial OR Epithelial Carcinoma, Ovarian OR Epithelial Carcinomas, Ovarian OR Ovarian Epithelial Carcinomas OR Epithelial Ovarian Cancer OR Ovarian Epithelial Cancer OR Cancer, Ovarian Epithelial OR Cancers, Ovarian Epithelial OR Epithelial Cancer, Ovarian OR Epithelial Cancers, Ovarian OR Ovarian Epithelial Cancers OR Ovarian Cancer, Epithelial OR Cancer, Epithelial Ovarian OR Cancers, Epithelial Ovarian OR Epithelial Ovarian Cancers OR Ovarian Cancers, Epithelial OR Ovarian Epithelial Carcinoma OR Epithelial Ovarian Carcinoma OR Carcinoma, Epithelial Ovarian OR Carcinomas, Epithelial Ovarian OR Epithelial Ovarian Carcinomas OR Ovarian Carcinoma, Epithelial) | 40372 |
| #2 | TS=(Fluorodeoxyglucose F18 OR F18, Fluorodeoxyglucose OR 18F-FDG OR Fluorodeoxyglucose F 18 OR F 18, Fluorodeoxyglucose OR Fludeoxyglucose F 18 OR F 18, Fludeoxyglucose OR Fluorine-18-fluorodeoxyglucose OR Fluorine 18 fluorodeoxyglucose OR 18F Fluorodeoxyglucose OR Fluorodeoxyglucose, 18F OR  18FDG OR 2-Fluoro-2-deoxy-D-glucose OR 2 Fluoro 2 deoxy D glucose OR 2-Fluoro-2-deoxyglucose OR 2 Fluoro 2 deoxyglucose) | 60678 |
| #3 | #1 AND #2 | 145 |

Ovid

| Search number | Query | Results |
| --- | --- | --- |
| #1 | (Carcinoma, Ovarian Epithelial or Carcinomas, Ovarian Epithelial or Epithelial Carcinoma, Ovarian or Epithelial Carcinomas, Ovarian or Ovarian Epithelial Carcinomas or Epithelial Ovarian Cancer or Ovarian Epithelial Cancer or Cancer, Ovarian Epithelial or Cancers, Ovarian Epithelial or Epithelial Cancer, Ovarian or Epithelial Cancers, Ovarian or Ovarian Epithelial Cancers or Ovarian Cancer, Epithelial or Cancer, Epithelial Ovarian or Cancers, Epithelial Ovarian or Epithelial Ovarian Cancers or Ovarian Cancers, Epithelial or Ovarian Epithelial Carcinoma or Epithelial Ovarian Carcinoma or Carcinoma, Epithelial Ovarian or Carcinomas, Epithelial Ovarian or Epithelial Ovarian Carcinomas or Ovarian Carcinoma, Epithelial).m_titl. | 19136 |
| #2 | (Fluorodeoxyglucose F18 or F18, Fluorodeoxyglucose or 18F-FDG or Fluorodeoxyglucose F 18 or F 18, Fluorodeoxyglucose or Fludeoxyglucose F 18 or F 18, Fludeoxyglucose or Fluorine-18-fluorodeoxyglucose or Fluorine 18 fluorodeoxyglucose or 18F Fluorodeoxyglucose or Fluorodeoxyglucose, 18F or 18FDG or 2 Fluoro 2 deoxyglucose or 2 Fluoro 2 deoxy D glucose or 2-Fluoro-2-deoxy-D-glucose or 2-Fluoro-2-deoxyglucose).m_titl. | 37289 |
| #3 | #1 AND #2 | 58 |

Pubmed

| Search number | Query | Results |
| --- | --- | --- |
| #1 | " Carcinoma, Ovarian Epithelial"[Mesh] | 6009 |
| #2 | (((((((((((((((((((((Carcinomas, Ovarian Epithelial[Title/Abstract]) OR (Epithelial Carcinoma, Ovarian[Title/Abstract])) OR (Epithelial Carcinomas, Ovarian[Title/Abstract])) OR (Ovarian Epithelial Carcinomas[Title/Abstract])) OR (Epithelial Ovarian Cancer[Title/Abstract])) OR (Ovarian Epithelial Cancer[Title/Abstract])) OR (Cancer, Ovarian Epithelial[Title/Abstract])) OR (Cancers, Ovarian Epithelial[Title/Abstract])) OR (Epithelial Cancer, Ovarian[Title/Abstract])) OR (Epithelial Cancers, Ovarian[Title/Abstract])) OR (Ovarian Epithelial Cancers[Title/Abstract])) OR (Ovarian Cancer, Epithelial[Title/Abstract])) OR (Cancer, Epithelial Ovarian[Title/Abstract])) OR (Cancers, Epithelial Ovarian[Title/Abstract])) OR (Epithelial Ovarian Cancers[Title/Abstract])) OR (Ovarian Cancers, Epithelial[Title/Abstract])) OR (Ovarian Epithelial Carcinoma[Title/Abstract])) OR (Epithelial Ovarian Carcinoma[Title/Abstract])) OR (Carcinoma, Epithelial Ovarian[Title/Abstract])) OR (Carcinomas, Epithelial Ovarian[Title/Abstract])) OR (Epithelial Ovarian Carcinomas[Title/Abstract])) OR (Ovarian Carcinoma, Epithelial[Title/Abstract]) | 14061 |
| #3 | #1 OR #2 | 16076 |
| #4 | " Fluorodeoxyglucose F18"[Mesh] | 34237 |
| #5 | ((((((((((((((F18, Fluorodeoxyglucose[Title/Abstract]) OR (18F-FDG[Title/Abstract])) OR (Fluorodeoxyglucose F 18[Title/Abstract])) OR (F 18, Fluorodeoxyglucose[Title/Abstract])) OR (Fludeoxyglucose F 18[Title/Abstract])) OR (F 18, Fludeoxyglucose[Title/Abstract])) OR (Fluorine-18-fluorodeoxyglucose[Title/Abstract])) OR (Fluorine 18 fluorodeoxyglucose[Title/Abstract])) OR (18F Fluorodeoxyglucose[Title/Abstract])) OR (Fluorodeoxyglucose, 18F[Title/Abstract])) OR (18FDG[Title/Abstract])) OR (2-Fluoro-2-deoxy-D-glucose[Title/Abstract])) OR (2 Fluoro 2 deoxy D glucose[Title/Abstract])) OR (2-Fluoro-2-deoxyglucose[Title/Abstract])) OR (2 Fluoro 2 deoxyglucose[Title/Abstract]) | 19183 |
| #6 | #4 OR #5 | 39905 |
| #7 | #3 AND #6 | 75 |
